# Obstetrical Statistics

**Published:** 1838

**Authors:** 


### Art. VI.—*Obstetrical Statistics.*

(Translated for the Western Journal, by J. A. W.)

Dr. Busch, professor of obstetricy in the University of Fred rick-William, at Berlin, has just published some statistics, comprising all the facts observed from the 1st of October 1829 to 31st December 1835.

During this time there has been 2056 births, of which 2035 were single and 21 double, so that the number of children is 2077

of the 2056 mothers, 992 were first deliveries, 1064 had been previously confined, 2 only were delivered after death; 2016 were discharged well, 30 died from the consequences of delivery. The youngest was 16, the oldest 45 years. Of the 2077 children, 1061 were boys, 1000 girls, sixteen the sex was not noted; 2045 were of full term; 32 before the time; 1945 were born alive, 132 still-born, 92 died during the month. 1911 were vertex presentations, 23, face, 28, feet, 2, knees, 47, breech, 54, altogether abnormal, 12 not observed.

Of the accouchments, 1711 were natural, 178 required the forceps, 4 had the natural position induced by external manipulations 57, were turned by the feet, 4, breech, 5, were forced labors, 3, premature, perforation was done 6 times, embryotomy twice, and the cesarean operation 3 times, once during the life of the mother.

The diseases of pregnancy were:—hypochondria 2, epilepsy 2, convulsions 2, intermittent fevers 4, dropsy 1, œdema of the feet 10, hœmoptysis, hernia, rheumatism, rheumatism of the uterus, continuation of the menses, menorrhagia, retroversion of the uterus, swelling of the neck of the uterus, cholera, each once.—*Rust's Magasin, No.* 49, 1837.

